# Reframing the role of glucagon‐like peptide 1 receptor agonists in cardiovascular medicine

**DOI:** 10.1002/ehf2.15123

**Published:** 2024-10-15

**Authors:** Riccardo M. Inciardi, Alvin Chandra, Ambarish Pandey, Marco Metra

**Affiliations:** ^1^ ASST Spedali Civili di Brescia, Department of Medical and Surgical Specialties, Radiological Sciences and Public Health University of Brescia Brescia Italy; ^2^ Division of Cardiology University of Texas Southwestern Medical Center Dallas Texas USA

In recent years, there has been growing evidence of the beneficial role of glucagon‐like peptide 1 receptor agonists (GLP1‐RA) in the treatment of obesity, type 2 diabetes (T2D) and prevention of cardiovascular (CV) disease.^1^, ^2^ Across large randomized clinical trials, GLP1‐RAs showed a 14% [hazard ratio (HR), 0.86; 95% confidence interval (CI), 0.80 to 0.93] risk reduction of major adverse cardiovascular events [MACE (myocardial infarction, stroke, or CV death)], 12% risk reduction of CV death, and 12% risk reduction of hospitalization for heart failure (HF) among patients with T2D.^1^ International guidelines have since recommended the use of GLP‐1 receptor agonists in patients with T2D with subclinical/clinical CV disease, overweight/obesity, or both.^3^

Recently, the role of GLP1‐RA has been supplemented by randomized clinical trials exploring the efficacy of semaglutide in both non‐diabetic and diabetic obese patients with HF with preserved ejection fraction (HFpEF) (the STEP‐HFpEF trial and STEP‐HFpEF DM trial, respectively)^4^ and obese patients with different CV risk profiles but without diabetes (the SELECT Trial).^5^ The STEP‐HFpEF trials enrolled HF patients with LVEF (left ventricular ejection fraction) ≥45% with and without diabetes and a body mass index (BMI) ≥30 kg/m^2^. Patients were randomized to receive 2.4 mg subcutaneous semaglutide once weekly or placebo.^4^ In the pooled analysis, semaglutide significantly improved in HF‐related symptoms (+7.5 points estimated treatment difference) and reduced body weight by ~8%. Although the trials were not designed to assess clinical events, there were fewer HF hospitalizations among the semaglutide‐treated patients as compared with placebo. The pooled analysis also showed robust safety data. Fewer serious adverse events, cardiac disorders and infectious disease disorders were recorded in the semaglutide group than in the placebo group. Gastrointestinal events leading to treatment discontinuation were more common in the semaglutide group than in the placebo group, although the frequency of serious gastrointestinal adverse events, including pancreatitis, was similar in both groups. It's important however to highlight that the trials enrolled selected patients and more data on safety events are needed from the general population.

In the Semaglutide Effects on Cardiovascular Outcomes in People with Overweight or Obesity (SELECT) trial,^5^ the administration of semaglutide (weekly subcutaneous dose of 2.4 mg) compared to standard care in patients with overweight or obesity and pre‐existing CV disease (82% with a history of coronary artery disease) led to a 20% risk reduction of a composite of death from CV causes, nonfatal MI, or nonfatal stroke (HR, 0.80; 95% CI, 0.72 to 0.90). Results are consistent with the previous SUSTAIN‐6 trial, which showed a 26% risk reduction (HR, 0.74; 95% CI, 0.58 to 0.95) of CV events in patients with diabetes treated with semaglutide 1 mg weekly.^6^ This is the first evidence of CV benefit derived using GLP1‐RA even among patients without T2D and the findings open new perspectives on the use of this class of drug in a broad context of CV prevention. Previous data suggested that the effect of GLP1‐RA may differ according to the HF phenotype with an attenuated effect among those with reduced LVEF, raising concerns about its use in this group of patients.^7^ Indeed, a secondary analysis of the FIGHT trial, showed that the safety profile of Liraglutide among patients with HFrEF was less pronounced.^8^ The SELECT trial shed new light in this context by enrolling more than 1300 patients with HFrEF. A prespecified analysis showed benefits of semaglutide in terms of major adverse CV events, HF events and mortality irrespective of investigator‐reported HF subtype.^9^ Yet, dedicated randomized trials are needed to clarify the efficacy on hard clinical endpoints and across the full spectrum of LVEF.^10^ The SELECT trial did not specifically include patients with a history of HF hospitalizations or elevated natriuretic peptides, potentially biasing the rate of clinical events compared to a dedicated randomized clinical trial and restricting the generalizability of this post hoc analysis.

Taken together, the STEP‐HFpEF and SELECT trials targeted a class of patients widely encountered in clinical practice in the US and Europe as millions suffer from obesity and HF with concomitant coronary artery disease and other CV risk factors/comorbidities. When compared to other lifestyle and pharmacologic interventions for overweight/obesity which did not show a clear benefit in terms of MACE risk reduction, semaglutide led to an early benefit of CV events suggesting that the magnitude of body‐weight loss may have mediated only part of the CV benefit. For instance, in the Harmony trial, albiglutide had a modest effect on glycaemic control and weight loss, but it was associated with a 22% reduction in CV events.^2^ A potential barrier in the medical therapy implementation of this drug may derive from the different formulation explored across trials. While subcutaneous semaglutide 1 mg weekly was effective among diabetic patients and recently among patients with T2D and chronic kidney disease in the FLOW trial,^11^ higher dosage (2.4 mg weekly) was explored among obese patients in the SELECT and STEP‐HFpEF programme. A recent meta‐analysis however showed across the SELECT, FLOW and STEP‐HFpEF programme, consistent favourable efficacy of semaglutide regardless of treatment regimens among HFpEF patients.^12^ Although oral semaglutide is also available, and other molecules are under development, most of the outcome data from clinical trials derive from the subcutaneous regimen. For instance, in the PIONEER 6 trial, oral semaglutide resulted in a significant weight loss, but was noninferior to placebo for CV outcomes or HF hospitalization outcomes in patients with T2D.^13^ More data are therefore needed to provide evidence of CV benefit from the oral administration as well.

**Figure 1:**
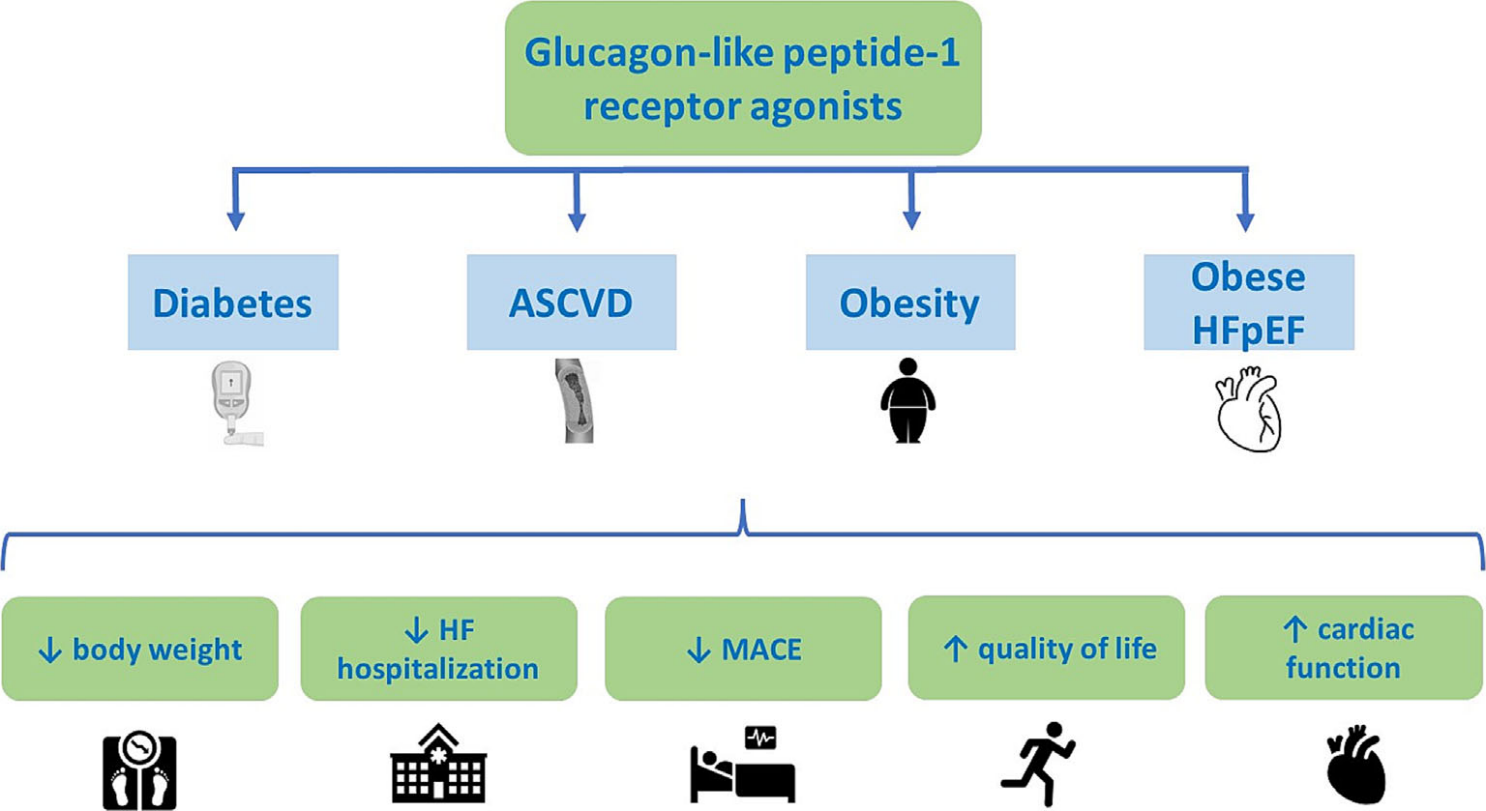
Cardiovascular benefit of glucagon‐like peptide 1 receptor agonists.

The exact underlying pharmacological mechanisms of GLP1‐RA, beyond body weight reduction, have not been entirely elucidated. Analysis from the STEP‐HFpEF programme showed that semaglutide compared with placebo consistently reduced NT‐proBNP and participants with higher baseline NT‐proBNP had a similar degree of weight loss but experienced larger reductions in HF‐related symptoms and physical limitations with semaglutide. Semaglutide also showed favourable effects to improve adverse cardiac remodelling in respect of left atrial volume, LV diastolic function and right ventricular size.^14^, ^15^ Taken together, these findings may suggest that the observed benefits of GLP1‐RA are unlikely to be simply related to weight loss but underlie specific disease‐modifying effects. The effects of GLP1‐RA are thought to contribute to inflammation reduction, endothelial and myocardial function improvement, promotion of atherosclerotic plaque stability and reduction of platelet aggregation.^2^, ^16^ GLP receptors are indeed expressed in the myocardium and the blood vessels. In this context, the receptor stimulation may increase cellular glucose uptake and improve LV function, may favour vasodilation, endothelial function improvement, inhibition of smooth muscle cells proliferation and increase in blood flow. Thus, these findings suggest a wide metabolic protective profile that may, at least in part, explain the CV benefit (*Figure* 1). Beyond available data on GLP1‐RA, the novel combined glucose‐dependent insulinotropic polypeptide (GIP‐RA) and GLP1‐RA tirzepatide will provide further pathophysiological evidence. Tirzepatide is the first approved dual GLP‐1 and GIP RA for glucose lowering in T2D and has indication for chronic weight management in adults with obesity or preobesity. The SURMOUNT Programme showed an overall weight reduction benefit from subcutaneous administration of tirzepatide ranging from 12% to 20% in obese patients with and without T2D.^17^ Finally, the SUMMIT trial (NCT04847557) recently showed a 38% risk reduction of HF (HF urgent visit or hospitalization, oral diuretic intensification or CV death), a 15.7% body weight reduction with improvement in health status among obese HFpEF (LVEF ≥ 50%) patients treated with tirzepatide compared to placebo. The ongoing SURPASS CVOT (NCT04255433) and SURMOUNT‐MMO (NCT05556512) will test the efficacy of this drug on major cardiovascular events in patients with and without diabetes, respectively.

The available results on the safety and efficacy of GLP1‐RA, must be considered in the context of a growing public health issue requiring the need to tackle the burden of atherosclerotic heart disease, obesity and HF regardless of the presence of diabetes. Despite the proven efficacy of directed medical therapy such as sodium‐glucose cotransporter 2 inhibitors, there is a significant residual burden of morbidity and mortality.^18^, ^19^ There is a need to implement novel therapeutic agents in this context along with other established preventive lifestyle^20^ and pharmacological interventions. Overall, the emergence of the GLP‐1 and GIP/GLP‐1 agonist offer significant opportunities to optimize metabolic health and symptom burden for patients with HF and obesity. In this context, lifestyle behaviour, including dietary modifications and physical activity, should form the bedrock of CV prevention and weight management plan and should be pursued even when a patient is treated with medications or surgical options. Obese patients with HF are at a high risk for polypharmacy, potentially enhancing the risk of drugs interaction and side effects. Also, initiation, up‐titration and monitoring of subcutaneous and oral GLP‐RA need to be carefully monitored especially to avoid gastrointestinal adverse effects. Given the multiple comorbidities and the complex management of these patients a multidisciplinary team that includes nurses, pharmacists, cardiologists, dietitians and diabetologists, appears critical in order to improve prevention and treatment strategies.

Medication access remains one of the most notable barriers to initiating novel pharmacotherapies. Yet, the high cost related to the use of this class of drug limits the accessibility to this treatment worldwide. Public policy and regulatory agencies are called for clinical care interventions to provide for social needs and access to treatments with GLP1‐RA. At the same time, future clinical trials are required to determine the efficacy of GLP1‐RA treatment among HF patients across the entire LVEF spectrum and among patients without T2D or obesity to further expand the indication of this class of drugs for the prevention of CV disease.
